# Fallopian tubes – literature review of anatomy and etiology in female infertility

**Published:** 2015

**Authors:** I Briceag, A Costache, VL Purcarea, R Cergan, M Dumitru, I Briceag, M Sajin, AT Ispas

**Affiliations:** * Department of Obstetrics and Gynecology, “Cantacuzino” Clinical Hospital, Bucharest, Romania; ** Ultrasound Teaching Center, “Carol Davila” University of Medicine and Pharmacy, Bucharest, Romania; *** Marketing and Medical Technology Department, “Carol Davila” University of Medicine and Pharmacy, Bucharest, Romania; **** Anatomy Department, “Carol Davila” University of Medicine and Pharmacy, Bucharest, Romania; ***** ”Carol Davila” University of Medicine and Pharmacy, Bucharest, Romania; ****** Pathology Department, “Carol Davila” University of Medicine and Pharmacy, Bucharest, Romania

**Keywords:** Fallopian, tubes, infertility, anatomy, etiology

## Abstract

**Rationale.** Around 30% of the infertile women worldwide have associated Fallopian tubes pathology. Unfortunately, for a long time, this aspect of infertility has been neglected due to the possibility of bypassing this deadlock through IVF.

**Objective.** Up to date free full text literature was reviewed, meaning 4 major textbooks and around 100 articles centered on tubal infertility, in order to raise the awareness on this subject.

**Methods and results.** The anatomy of the Fallopian tube is complex starting from its embryological development and continuing with its vascular supply and ciliated microstructure, that is the key to the process of egg transport to the site of fertilization. There are many strongly documented causes of tubal infertility: infections (Chlamydia Trachomatis, Gonorrhea, and genital tuberculosis), intrauterine contraceptive devices, endometriosis, and complications after abdominal surgery, etc.

**Discussions.** Although there are still many controversies about the etiology of tubal sterility with the advent of molecular diagnosis of infections there has been cleared the pathway of infection through endometriosis or through ciliary immobility towards the tubal obstruction.

## Introduction

In order to suspect infertility a patient should present with failure to achieve a successful pregnancy after 12 month or more of regular unprotected intercourse in a woman under the age of 35 years and 6 months without success in a woman 35 or older [**1**]. WHO estimated that infertility affects 50-80 million women worldwide and 11.3% of married women with only 35% of these presenting for medical help [**2**]. Tubal subfertility or infertility is credited with up to 30% of the etiology of infertility [**3**]. Bypassed by in vitro fertilization (IVF) the fallopian tube has until recently been denied its role as the site of fertilization and early embryogenesis that can be easily disrupted by common conditions such as Chlamydia trachomatis infections [**4**]. While research and clinical experience continue to indicate that the vast majority of infertile men and women do not experience significant levels of psychological trauma, the use of advanced medical technology and third party reproduction may increase psychological distress during specific periods of treatment [**5**].

## Methods

We queried international database for published works on the subject of tubal infertility using the key words fallopian and tubal and infertility. Therefore we have identified 20 major textbooks on the subject of infertility, but only 4 were fully dedicated to this subject, the oldest published in 1988 by Hunter RHF et al, and the newest in 2010 by Ledger WL, et al. There are 461 free full text articles on the subject of infertility but only 104 are centered on the etiology, diagnosis and management of tubal infertility. The number of articles per central theme was distributed as following: 4 generic articles on this subject, 6 on anatomy, 28 about the etiology, 48 on diagnosis modalities, and 18 about the treatment options.

## Results

**Fig. 1:**
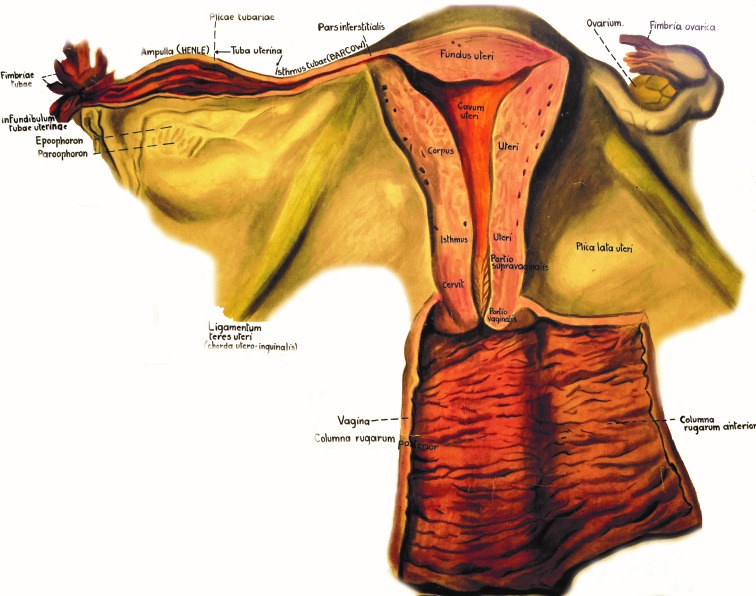
Anatomy of the female reproductive system – chart taken and modified from the collection of the Anatomy Department, “Carol Davila” University of Medicine and Pharmacy, Bucharest

The Fallopian tubes are muscular conduits connecting the ovaries with the uterus and are divided into the following regions: fimbriated infundibulum, ampulla and isthmus (**Fig. 1**). At puberty, the extra-uterine portion of the tube measures approximately 11cm and the intra-mural portion is 1.5-2cm long, these dimensions are concealed by the convolutions of the duct in situ within its supporting mesentery, the mesosalpinx [**6**]. 

**Fig. 2:**
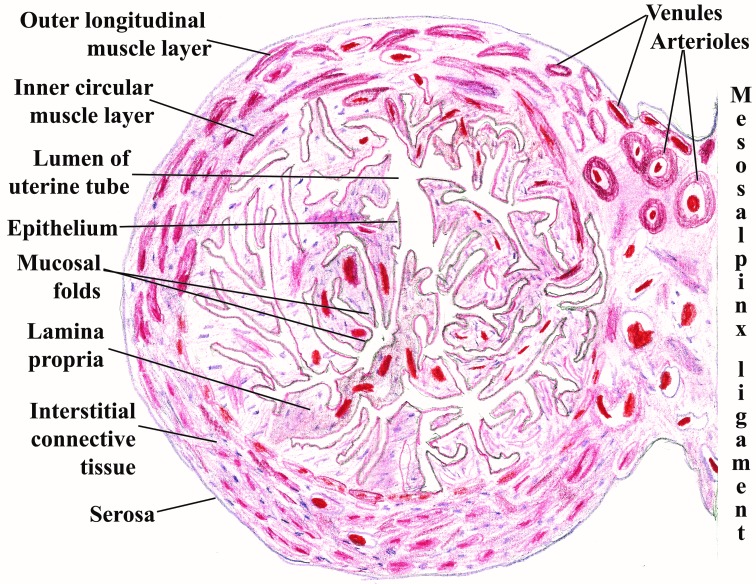
Uterine tube at the level of the ampulla and mesosalpinx ligament. Schematic drawing after a hematoxylin and eosin specimen viewed at low magnification from the collection of the Morphology Department at “Carol Davila” University of Medicine and Pharmacy, Bucharest

Each of these three divisions of the Fallopian tubes presents characteristic histological features that underline their different physiological functions. For example, in a study on 30 female patients, Neamtu MC, et al. [**7**] have observed the stereo distribution of myometrial vascular units at the level of the uterine-tubal junction, explaining the existence of a plexiform network of cavernous type, which has a rich ortho-sympathetic vegetative innervations responsible for the unfavorable evolution of myometrial circulatory system postpartum (**Fig. 2**).

The female genital tract arises from the Mullerian duct system due principally to the elaboration of the paramesonephric ducts. From the proximal portion of the Mullerian duct through a complex dynamic process evolves the functional fallopian tube [**8**].

The arterial supply of the tubes is derived from both the uterine and ovarian arteries and is subject to changes synchronous to menstrual cycle and the different stages of pregnancy. The venous return closely follows the arteries with interconnecting capillary networks beneath the serosa, in the muscular layer and in the mucosa. Isaksson R, et al. [**9**] compared the uterine and spiral artery blood flow in women with unexplained and tubal infertility discovering virtually no difference in the context of local counter-current transfer systems.

The inner surface of the epithelium of the tubes is lined by ciliated cells which are most prominent at the level of the fimbriated infundibulum where they form dense arrays. Moreover, the ciliary activity is vigorous in the secretory phase of the menstrual cycle. Numerous pathological conditions associated with infertility have been proven to destroy cilia or to reduce ciliary motion [**10**].

## Discussions

The anomalies of the tubes range from congenital absence [**11**], major diverticula, duplication of the tubes [**12**] to occlusion at all levels with or without hydrosalpinx.

Etiology of tubal infertility: infections (Chlamydia trachomatis, Gonorrhea, genital tuberculosis), intrauterine contraceptive devices, endometriosis, complications after abdominal surgery [**13**].

Age is also important as the adjusted odds (95% CI) of having a diagnosis of tubal factor infertility at 35-39 years were 2.2 (1.7-2.7) as compared to a woman less than 30 [**14**].

Subfertile women IgG-positive for C. trachomatis with raised CRP concentrations have persistent infections and are at highest risk of tubal infertility [**15**].

Concerning genital tuberculosis it has been proven that PCR testing enables earlier diagnosis than conventional methods: histopathological examination, microscopic examination of acid fast bacilli and mycobacterium culture [**16**].

One of the most recent studies concerning the connection between abdominal surgery and tubal infertility was performed by Ramurewa O, et al. [**17**] on 130 patients with proven tubal infertility.

The use of intrauterine contraceptive devices is linked to tubal subfertility only when the procedure is accompanied by infections in the first 20 days after the insertion [**18**].

Proliferative endometrial development and formation of endometrial polyps predispose to the formation of similar cornual polyps [**19**].

## Conclusions

As in many other fields after the extensive use of hysterosalpingography and laparoscopy prior to in vitro fertilization techniques now we notice a back to basics turn in the focus of worldwide research community studying the tubal infertility. After an extensive knowledge of normal anatomy and its variants, the fertility specialist should master novel serological techniques in the detection of pelvic inflammatory disease along with the concept of ciliary movement impairment. Therefore, one must be a little more reserved facing a normal hysterosalpingography as this does not mean always a clear passage for the egg and subsequent fertilization. As a paradox, these new findings question the efficacy of current surgical procedures addressing the tubal infertility and put more pressure on IVF techniques.

**Disclosures**

None
